# Clinical registry of dental outcomes in head and neck cancer patients (OraRad): rationale, methods, and recruitment considerations

**DOI:** 10.1186/s12903-017-0344-y

**Published:** 2017-02-27

**Authors:** Rajesh V. Lalla, Leslie Long-Simpson, James S. Hodges, Nathaniel Treister, Thomas Sollecito, Brian Schmidt, Lauren L. Patton, Michael T. Brennan, for the OraRad Study Group

**Affiliations:** 1 Section of Oral Medicine, MC1605, University of Connecticut Health, 263, Farmington Avenue, Farmington, CT 06030-1605 USA; 2 Division of Biostatistics, School of Public Health, University of Minnesota, 2221 University Ave SE Suite 200, Minneapolis, MN 55414 USA; 3 Division of Oral Medicine and Dentistry, Brigham and Women’s Hospital, 1620 Tremont Street, 3rd Floor, Boston, MA 02120 USA; 4 Department of Oral Medicine, Infection and Immunity, Harvard School of Dental Medicine, 188 Longwood Avenue, Boston, MA 02115 USA; 5 Department of Oral Medicine, University of Pennsylvania School of Dental Medicine, 240 South 40th Street, Philadelphia, PA 19104 USA; 6 Department of Oral & Maxillofacial Surgery and Bluestone Center for Clinical Research, New York University College of Dentistry, 421 First Avenue, New York, NY 10010 USA; 7 Department of Dental Ecology, School of Dentistry, University of North Carolina, Chapel Hill, NC CB 7450 USA; 8 Department of Oral Medicine, Carolinas Medical Center, 1000 Blythe Blvd, Charlotte, NC 28203 USA

**Keywords:** Head and neck cancer, Radiation therapy, Protocol, Tooth loss, Exposed bone, Osteoradionecrosis, Salivary flow, Periodontal disease, Caries, Recruitment

## Abstract

**Background:**

Most head and neck (H&N) cancer patients receive high-dose external beam radiation therapy (RT), often in combination with surgery and/or chemotherapy. Unfortunately, high-dose RT has significant adverse effects on the oral and maxillofacial tissues, some of which persist for the life of the patient. However, dental management of these patients is based largely on individual and expert opinion, as few studies have followed patients prospectively to determine factors that predict adverse oral sequelae. In addition, many previous studies were conducted before wide-spread adoption of intensity-modulated radiation therapy (IMRT) and concurrent chemotherapy. The objective of this multi-center study is to systematically evaluate the oral health of subjects for 2 years after commencement of RT, with the goal of identifying risk factors that predict adverse oral outcomes post-RT.

**Methods:**

This is a prospective multi-center longitudinal cohort study of H&N cancer patients who receive high-dose RT with curative intent. Planned enrollment is 756 subjects at 6 primary clinical sites (and their affiliated sites) in the USA. A baseline visit is conducted prior to the beginning of RT. Follow-up visits are conducted at 6, 12, 18 and 24 months from the start of RT. The primary outcome measure is the 2-year rate of tooth loss in patients who have received at least one session of external beam RT for H&N cancer. Secondary outcome measures include the incidence of exposed intraoral bone; incidence of post-extraction complications; change in Decayed Missing and Filled Surfaces (DMFS); change in periodontal measures; change in stimulated whole salivary flow rates; change in mouth opening; topical fluoride utilization; chronic oral mucositis incidence; changes in RT-specific quality of life measures; and change in oral pain scores.

**Discussion:**

This study will contribute to a better understanding of the dental complications experienced by these patients. It will also enable identification of risk factors associated with adverse outcomes such as tooth loss and osteoradionecrosis. These findings will support the development of evidence-based guidelines and inform the planning of future interventional studies, with the goal of advancing improvements in patient care and outcomes.

**Trial registration:**

ClinicalTrials.gov Identifier NCT02057510, registered 5 February 2014.

## Background

Most head and neck (H&N) cancer patients receive high-dose external beam radiation therapy (RT), often in combination with surgery and/or chemotherapy [1]. Unfortunately, high-dose RT has significant adverse effects on the oral and maxillofacial tissues, some of which persist for the life of the patient [2]. The salivary glands are often included in the fields of radiation, which frequently leads to irreversible hyposalivation [3], resulting in increased rates of dental caries and tooth loss post-radiation [4]. Radiation-induced soft tissue fibrosis can lead to restricted mouth opening (trismus), which reduces the ability to maintain oral hygiene [5]. Furthermore, radiation impairs bone homeostasis and ability to heal, leading to a life-long risk of jaw osteoradionecrosis (ORN), which is often precipitated by dental extractions [6]. Consequently, these patients are caught in a vicious circle, being at higher risk of needing dental extractions, but with extractions to be avoided post-RT due to the risk for ORN.

To attempt to reduce the frequency of such complications, pre-RT dental evaluation and management is considered a best practice and standard of care in the USA and many other countries. However, pre-RT dental management practices vary widely among centers, due to a lack of reliable data on dental morbidity in these patients and on outcomes of dental management strategies. Although some studies indicate that these patients have significant dental morbidity post-RT, definitive data to document the extent, severity and risk factors for these complications do not exist. Consequently, pre-RT dental management is largely based on expert opinion [7]. For example, an international survey of dentists/oral surgeons experienced in providing pre-RT care found that approximately 50% would extract a mandibular tooth in the radiation field with a small periapical lesion and pain on percussion, while 50% would treat the tooth endodontically [8]. Clinicians mentioned that the lack of adequate data reduced their confidence in their treatment decisions. Such disparate decision-making can result in sub-optimal dental and medical outcomes for these complex patients.

The objective of this prospective observational cohort study is to collect data on dental outcomes in patients who have received RT to the H&N region. Systematic collection of this data in a large multi-center cohort will lead to a better understanding of the dental complications experienced by these patients. In addition, it will enable the identification of risk factors associated with negative outcomes such as tooth loss and ORN. These findings will support the development of evidence-based guidelines and should lead to improvements in patient care and outcomes. The purpose of this manuscript is to describe the study protocol (version 4, 29 October 2015) for this important ongoing study and provide insight based on our experience related to subject recruitment and complexity of study procedures.

## Methods

### Study design

This is a prospective multi-center longitudinal cohort study of H&N cancer patients who receive high-dose RT.

### Outcome measures

The primary outcome measure is the 2-year rate of tooth loss in patients who have received at least one session of external beam RT with curative intent for H&N cancer.

“Tooth loss” is defined as a dental extraction that has been performed or recommended. Teeth that are lost without the involvement of a healthcare professional are also included in this definition. Since dental extractions are often avoided in this population because of the increased risk of ORN, tooth loss also includes teeth having a dental procedure to avoid extraction of a tooth that would have been extracted if the individual had not received RT. The following categories constitute teeth that would otherwise be recommended for extraction: non–restorable because of fracture or extent of caries; amputated crown with root remaining; uncontrolled or persistent periodontal or odontogenic infection. Such teeth are classified as “hopeless teeth” for the purposes of this study.

Secondary outcome measures include the following additional oral health outcomes associated with RT in H&N cancer patients and potential risk factors for negative outcomes:

- Incidence of exposed intraoral bone, suggestive of ORN. This will be defined as exposed maxillary or mandibular bone with an avascular appearance in a quadrant that has received RT;
- Incidence of post-extraction complications;
- Change in Decayed, Missing, and Filled Surfaces (DMFS);
- Change in periodontal measures;
- Change in stimulated whole salivary flow rates;
- Change in mouth opening in mm;
- Use of fluoride to prevent new caries;
- Incidence of chronic oral mucositis;
- Change in RT-specific quality of life measures; and
- Change in pain scores as measured with the UCSF Oral Cancer Pain Scale.

### Study organization and funding

Subjects are enrolled at six primary clinical study sites, which are (in alphabetical order): Brigham and Women’s Hospital, Carolinas Medical Center, New York University, University of Connecticut Health, University of North Carolina, and University of Pennsylvania. Personnel at each site include the site Principal Investigator (PI), study coordinator(s), and clinical examiner(s). Some of the primary clinical sites have also established local affiliated sites. The Data Coordinating Center (DCC) for the study is located at the University of Minnesota (Division of Biostatistics) and includes the site PI/study statistician, study manager, and database administrator. The study is funded by the US National Institute for Dental and Craniofacial Research (NIDCR) through a cooperative agreement (U01) mechanism. NIDCR staff are closely involved in the development and oversight of the study.

### Safety of human subjects and data integrity

Approval from the Institutional Review Board (IRB) at each clinical study site was obtained before beginning enrollment at that site. Any changes are also communicated to IRB and other stakeholders as appropriate. In addition, each site was individually activated by the NIDCR after passing all relevant requirements for human subjects safety and personnel training. All subjects participate in an informed consent process conducted by the study coordinator, including the signing of a written informed consent document. An independent Data and Safety Monitoring Board (DSMB) has been constituted by the NIDCR to ensure subject safety. It has 3 members, all of whom are free from competing interests. It meets annually and additional meetings can be called as needed. The DSMB reviews any adverse events as well as other study data. No safety concerns have been identified to date.

Study data is entered into a secure database, hosted at the DCC, using web-based data-entry screens created for this study. DCC staff monitor study data to identify missing data or forms and communicate with the clinical sites to resolve these issues. Formal monitoring of study records is conducted by an independent contract research organization. Study Monitors visit each clinical site annually for formal study monitoring.

### Training and calibration

All study personnel receive training on the activities they conduct in the study. This includes training on carrying out clinical assessments as well as training on completing study forms, data entry, and all non-clinical procedures. In addition, annual in-person calibration on healthy volunteers is conducted for all clinical examiners for DMFS and periodontal measurements. Clinical examiners must meet minimum standards for intra- and inter-rater consistency annually for these measurements. Specifically, examiners must achieve at least 95% intra-examiner reproducibility within ± 2 mm for both clinical attachment loss and pocket depth; at least 75% inter-examiner agreement for pocket depth within ± 1 mm; and at least 60% inter-examiner agreement for clinical attachment loss within ± 1 mm. Examiners also receive hands-on training on measuring furcation involvement and tooth mobility.

### Subject selection criteria

#### Inclusion criteria

To be eligible to participate in this study, a subject must meet all of the following criteria:

- Diagnosed with H&N squamous cell carcinoma (SCC) or a salivary gland cancer (SGC), and intends to receive external beam RT with curative intent (tumor eradication), with or without concomitant chemotherapy;
  OR
  Diagnosed with a non-SCC, non-SGC malignancy of the H&N region, and intends to receive RT, with or without concomitant chemotherapy. The subject must be expected to receive at least 4500 cGy to at least 1 of 26 specified sites in the H&N region;
- Aged 18 years and older;
- Willing and able to provide signed and dated consent form;
- At least 1 natural tooth remaining or expected to remain in the mouth after completion of the pre-RT dental extractions, if any;
- Willing to comply with all study procedures; and
- Willing to participate for the duration of the study.

After the completion of the baseline study visit, it must be verified that the subject has received at least one session of RT, to confirm eligibility for continued follow-up in the study.

#### Exclusion criteria

A potential subject who meets any of the following criteria will be excluded from participation in this study:

- Receiving palliative RT;
- History of prior curative RT to the H&N region to eradicate a malignancy;
- Incarcerated at time of screening; or
- Anything that would place the subject at increased risk or preclude the subject’s full compliance with or completion of the study.

### Study assessments

**Fig. 1:**
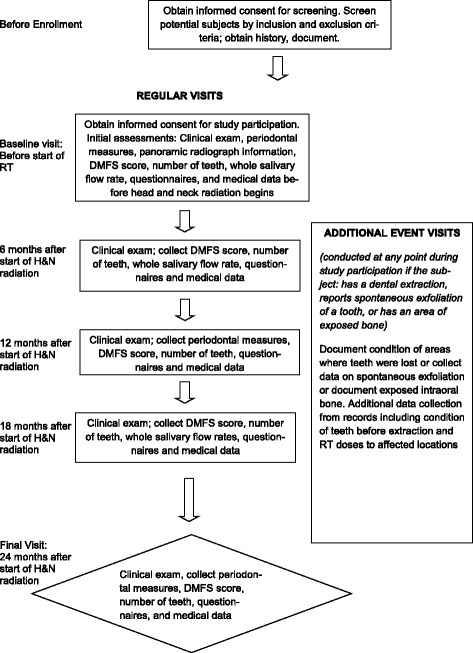
Sequence of a subject’s participation

Figure 1 depicts the sequence of events in a subject’s participation. A baseline visit is conducted before the beginning of RT but after completing any recommended pre-RT extractions and other invasive oral procedures. Follow-up visits are conducted at 6, 12, 18, and 24 months (+/- 30 days) from the start of RT. Table 1 summarizes the procedures conducted at each study visit.

**Table 1:** Study Procedures at each time-point (per SPIRIT guidelines)

Procedures		Screening	Baseline Visit (0 – 42 days before RT begins)	Baseline records review after RT	Follow-up Visit 1 (182 ± 30 days after RT begins)	Follow-up Visit 2 (364 ± 30 days after RT begins)	Follow-up Visit 3 (546 ± 30 days after RT begins)	Follow-up Visit 4 (728 ± 30 days after RT begins)	Early Withdrawal Visit	Unscheduled Visit
Consent for screening, assess eligibility, record basic demographic data		X								
Obtain signed study consent form, confirm eligibility			X							
Review medical and dental records	Diagnosis and treatment plan	X	X							
	Medication use		X		X	X	X	X	X	
	Pre-RT dental care delivered			X						
	Data on extractions and complications		X		X	X	X	X	X	
	Total amount of radiation received			X						
Oral/Dental Assessment	DMFS		X		X	X	X	X	X	
	Number of teeth, removable prostheses		X		X	X	X	X	X	
	Presence of intrabony devices		X							
	Periodontal measures^a^		X			X		X	X^c^	
	WHO oral mucositis scale		X		X	X	X	X	X	
	Mouth opening measurement		X		X	X	X	X	X	
	Panoramic radiograph^b^		X							
	Tooth loss, ORN, dental infection/complication		X		X	X	X	X	X	
Measure salivary flow rate			X		X		X		X^c^	
Obtain participant-reported data	Demographics, tobacco and alcohol use		X							
	Fluoride use and oral hygiene practices		X		X	X	X	X	X	
	Oral cancer pain assessment		X		X	X	X	X	X	
	Oral health-related quality of life (RT-specific)		X		X	X	X	X	X	
Post-extraction/exfoliation/exposed bone assessment (if applicable)			X		X	X	X	X	X	X

^a^Periodontal measures at baseline include clinical attachment loss, bleeding on probing, pocket depth, gingival recession, plaque index, tooth mobility and furcation involvement; at follow-up visits, plaque index, tooth mobility and furcation involvement are not recorded

^b^Findings from clinical radiograph are recorded; radiograph is taken for study only if a current (<180 days) radiograph from routine clinical care is not available

^c^Measured at early withdrawal visit only if it would have been done at next scheduled visit

Details of study assessments, including instruments/scales used, are as follows:

- A panoramic radiograph taken within 180 days before the baseline study visit is evaluated to assess the presence of intrabony devices, periapical lesions and impacted teeth.
- Periodontal measures are collected at baseline, 12, and 24 month visits. Probing depths and the distance from the cemento-enamel junction to the gingival margin are measured using a UNC 15 probe at six sites on each tooth. The clinical attachment level is calculated by the DCC using these two recorded measurements. The presence of bleeding upon probing is documented at the six periodontal probing sites. These assessments are obtained on all teeth other than third molars. Additionally, at the baseline visit, a plaque score is recorded for the Ramfjord teeth, furcation involvement is measured using a Naber’s probe, and tooth mobility is assessed [9]. The invasive periodontal measures are not performed on patients needing antibiotic prophylaxis prior to invasive dental procedures.
- Decayed, missing, filled surfaces (DMFS) are recorded at baseline, 6, 12, 18, and 24 month visits. The dentition is examined for active decay, missing teeth because of extraction and/or spontaneous exfoliation, and teeth that have restorations. A No. 23 explorer or a No. 2A explorer is used to detect decay. Five tooth surfaces on the posterior teeth and four tooth surfaces on the anterior teeth are scored for DMFS. The DMFS is also used to reconcile newly missing teeth between study visits.
- Salivary flow rate is collected at baseline, 6, and 18 month visits. Participants are provided with unflavored paraffin (gum base) and two 50 ml test tubes. They are instructed to chew the gum base for 2 min, meanwhile expectorating saliva into one of two test tubes. This is done as practice to standardize the technique and stabilize the flow rate. The same chewing/expectorating method is used for 5 min for the final flow rate assessment, timed using a digital timer. The saliva collected in 5 min is weighed and recorded.
- Additional assessments completed at all visits include clinical examination of the subject’s oral cavity for “hopeless teeth” (using criteria described above), grading of oral mucositis using the World Health Organization (WHO) mucositis scale [10], inspection of both the mandible and maxilla for exposed intraoral bone, and measurement of maximal mouth opening using a Therabite® Range of Motion Scale in millimeters.
- Patient-reported questionnaires are administered at all study visits. These questionnaires collect information on the participant’s daily oral hygiene habits, oral pain using the UCSF Oral Cancer Pain Scale [11], and quality of life related to their oral health using items from the EORTC QLQ-H&N35 scale [12].
- Additional “Event visits” are conducted if a subject has any of the following “events”: has a dental extraction, reports spontaneous exfoliation of a tooth, or has an area of exposed intraoral bone. Any signs and symptoms the subject may present with at the visit are recorded, including those related to infection and wound healing. Additional data, related to the event, is collected from the medical and dental records, including radiation doses to affected locations and the condition of the teeth before extraction.

### Statistical considerations

#### Sample size and power considerations

Total planned enrollment is 756 subjects across all clinical sites. The primary outcome used to determine sample size is the rate of tooth loss after the end of RT. Although ORN is an important outcome in these patients, the relative infrequency of ORN [13] makes it unfeasible to conduct a study with ORN as a primary outcome measure. Tooth loss is an ideal outcome measure because it is more common than ORN, it is easily measured, and ORN in this population often results from post-RT dental extractions.

Previous studies found that 20 of 168 patients either had or were recommended to have a dental extraction after median follow-up of 7.6 months after end of H&N RT [14], and that 10 of 50 patients (20%) reported having had an extraction or a procedure to avoid an extraction at a median follow-up of 728 days after the end of RT, with median time of 442 days from the end of RT to the procedure (Unpublished data, University of Connecticut). We also estimated that up to 30% of enrolled participants would withdraw from study participation, be lost to follow-up, or become deceased during 2 years of follow-up. Based on the foregoing, we assumed 20% of subjects would have an extraction or a procedure to avoid an extraction by 2 years after RT, so that 756 enrolled subjects would yield 529 participants with 2-year follow-up data on tooth loss and an expected 95% confidence interval (around 20%) of 16.7%–23.7%, which we deemed adequately narrow. If the actual rate of tooth loss is lower than 20%, a 95% confidence interval will be narrower; if the actual rate of tooth loss is higher than 20%, a 95% confidence interval will be wider. Under these assumptions, 88–125 subjects will have a tooth-loss event. Based on common rules of thumb, this suffices to consider 8–12 predictors (risk factors) for tooth loss [15].

#### Planned analyses

The study’s primary objective is descriptive so the analyses are mainly descriptive. The primary objective can be interpreted in two ways: What fraction of subjects had a tooth-loss event in the two years after RT? And how many teeth were lost per year? Analyses of the former will use time-to-event (survival) methods, where the event is the first tooth loss. This type of analysis accounts for partial follow-up. The 2-year fraction with tooth-loss events will be estimated using the survivor function and associated confidence interval. To estimate the rate of teeth lost per year, each subject’s outcome is the number of teeth they lost, analyzed using a negative binomial model with log link and offset log follow-up time (in years) and no predictors. This model’s intercept, raised to the power e (the base of the natural logarithm), estimates the rate of teeth lost per year. (The negative binomial model allows analysis of a count outcome with a flexible error variance. Obviously this model is inappropriate if many participants lose large numbers of teeth but this seems unlikely.) Secondary analyses will cull potential risk factors for tooth loss events and for increased rate of tooth loss by considering them as predictors in the regression analyses described above. Other secondary analyses will consider the secondary outcomes, many of which are measured at multiple follow-up visits, so their analyses will be longitudinal, accounting for the presumed correlation of a subject’s multiple measurements.

One complication of these analyses is how a subject’s initial number of teeth affects tooth loss. We will examine this question in preliminary analyses that include in the regression a flexible function of the initial number of teeth (e.g., a 4°-of-freedom spline). A further complication arises from subjects who have incomplete follow-up due to death. We account for this by defining the outcome being estimated as “rate [or chance] of tooth loss assuming survival to 2 years”, which can be estimated by various methods that accommodate participants with incomplete follow-up, e.g., inhomogeneous Poisson processes or logistic regression including a flexible function of follow-up time as an adjuster.

## Discussion

The results of this ongoing study are expected to be valuable for several reasons. First, this study will definitively document the oral and dental morbidity resulting from high-dose RT to the H&N region. This includes the impact on salivary flow, mouth opening, caries, periodontal disease, tooth loss, and development of ORN. A better understanding of these downstream effects of RT will inform the pre-RT dental evaluation and reinforce its importance to all stakeholders including patients, medical and dental providers, and third-party payers. Second, this study should allow the identification of risk factors for adverse oral and dental outcomes. As mentioned previously, the pre-RT dental management of these patients varies widely and is largely based on expert opinion. Identification of specific risk factors will allow for evidence-based decision making and better clinical outcomes. Thus, our findings have the potential to lead to significant changes in clinical practice. Third, this study provides a unique opportunity to compare pre-RT dental management practices across a number of different sites. An assessment of important factors, such as number of pre-RT dental extractions, duration between extractions and start of RT, and subsequent development of ORN, in this large cohort can be highly informative.

There are some limitations of this study design. Two years is a relatively short follow-up period as tooth loss may occur after this time. While a longer follow-up period would be desirable, funding constraints did not allow for this and it can also be challenging to follow such medically complex patients for longer periods. Important assessments such as periodontal measurements and salivary flow are not conducted at each follow-up visit. These are completed at alternating follow-up visits to reduce the length of the follow-up visits and burden on the subject. Although the study Manual of Procedures recommends that each subject should be examined by the same clinical examiner at all visits, in practice this has been difficult to achieve due to scheduling issues and staff turnover. However, all clinical examiners are calibrated to strict standards for inter- and intra-rater consistency.

Recruitment presents a challenge in most clinical studies and especially in the oncology population. It has been estimated that only about 3% of patients with cancer participate in a clinical trial and even at some tertiary academic centers, the rate may be below 10% [16]. A barrier to recruitment for the present study is that in order for the baseline visit to be conducted prior to the start of RT, patients may need to be approached soon after their cancer treatment plan has been determined. At this time, patients are often emotionally fragile and overwhelmed with details from various providers on planned surgery, chemotherapy, and RT. In the context of a life-threatening diagnosis, patients can be bewildered about being approached for participation in an observational dental study that does not offer any direct impact on their cancer prognosis. Indeed, as of December 2015, the most common reason (60%) for an approached patient not being formally screened is that they are “not interested/too busy”. Careful and patient explanation of the dental and oral complications of RT, and possible benefits of study participation, can be helpful, particularly if reinforced by the radiation oncologist. It is highly desirable to have a team-based approach, where all parties recognize and reinforce the importance of the study and benefits of participation, to the subject and to society. Potential benefits to the subject include the regular and detailed monitoring of their oral and dental status at no charge, which can facilitate the earlier diagnosis and management of potential oral complications. Appropriate monetary compensation is also provided to subjects for their time and effort in attending study visits. However, the study does not directly provide or support clinical care, which may have a negative effect on some patients’ interest in study participation.

An important aspect of recruitment is access to the patient population. It is standard practice in the USA for these patients to be referred to a dentist for a pre-RT dental assessment [1]. This dental assessment may occur at the cancer treatment site itself or at a community dental practice. Our experience at the different clinical sites has shown that recruitment is enhanced when the study team works closely with the clinical dental providers receiving the clinical referral and/or the oncology team managing the medical care of these patients. However, the pre-RT clinical dental evaluation can also be a reason patients decline study participation. Specifically, when the pre-RT dental evaluation by regular clinical providers includes periodontal probing, patients have sometimes been unwilling to then undergo the additional detailed periodontal examinations conducted at the baseline study visit by the calibrated study examiner.

The number of eligible patients also varies across sites, with sites located in large urban centers or tertiary care centers usually having a larger patient population. Indeed, such higher profile referral centers have been the strongest enrolling sites. However, a proportion of patients at such magnet referral centers travel from a distance for their cancer care but may receive follow-up closer to home. This can lead to sub-optimal compliance with the subsequent follow-up visits after treatment. Thus, appropriate selection of patients is important. The main barrier to recruitment at smaller centers is access to an adequate number of eligible patients. The smaller sites, however, also tend to have better attendance at follow-up visits. Strategies we have adopted at sites with lower enrollment include the establishment of a satellite study location at a nearby healthcare facility, where enrollment activities and/or study visits can take place. While this has had a modest positive impact on enrollment, it does create new challenges in terms of obtaining medical records from the satellite site.

An additional factor affecting recruitment is adequate staffing, given the ongoing demands related to scheduling and conducting follow-up visits as well as data management. To better optimize use of the study staff’s time, the amount of medications data being collected at follow-up visits was significantly reduced after about 250 subjects had been enrolled. An analysis of the medications data collected to that point revealed that, beyond the 6 month visit, very few subjects were using the medications being collected. Therefore, collection of detailed medications data from medical records after that point was determined as not being critical to achieve the main study objectives.

Notwithstanding all of the above-mentioned recruitment considerations, over 450 subjects have been enrolled to the study so far. Thus, this landmark study is expected to meet its planned enrollment target and result in significant advances in the care of patients receiving RT to the H&N region.
